# MUC1 Expression Affects the Immunoflogosis in Renal Cell Carcinoma Microenvironment through Complement System Activation and Immune Infiltrate Modulation

**DOI:** 10.3390/ijms24054814

**Published:** 2023-03-02

**Authors:** Giuseppe Lucarelli, Giuseppe Stefano Netti, Monica Rutigliano, Francesco Lasorsa, Davide Loizzo, Martina Milella, Annalisa Schirinzi, Antonietta Fontana, Francesca Di Serio, Roberto Tamma, Domenico Ribatti, Michele Battaglia, Elena Ranieri, Pasquale Ditonno

**Affiliations:** 1 Urology, Andrology and Kidney Transplantation Unit, Department of Precision and Regenerative Medicine and Ionian Area, University of Bari “Aldo Moro”, 70124 Bari, Italy; 2 Molecular Medicine Center, Section of Clinical Pathology, Department of Medical and Surgical Sciences, University of Foggia, 71122 Foggia, Italy; 3 Clinical Pathology Unit, Polyclinic University Hospital, 70124 Bari, Italy; 4 Department of Translational Biomedicine and Neurosciences, University of Bari “Aldo Moro”, 70124 Bari, Italy

**Keywords:** renal cell carcinoma, MUC1, PTX3, CA15-3, kynurenine, complement, immune cell

## Abstract

Mucin1 (MUC1), a glycoprotein associated with an aggressive cancer phenotype and chemoresistance, is aberrantly overexpressed in a subset of clear cell renal cell carcinoma (ccRCC). Recent studies suggest that MUC1 plays a role in modulating cancer cell metabolism, but its role in regulating immunoflogosis in the tumor microenvironment remains poorly understood. In a previous study, we showed that pentraxin-3 (PTX3) can affect the immunoflogosis in the ccRCC microenvironment by activating the classical pathway of the complement system (C1q) and releasing proangiogenic factors (C3a, C5a). In this scenario, we evaluated the PTX3 expression and analyzed the potential role of complement system activation on tumor site and immune microenvironment modulation, stratifying samples in tumors with high (MUC1H) versus tumors with low MUC1 expression (MUC1L). We found that PTX3 tissue expression was significantly higher in MUC1H ccRCC. In addition, C1q deposition and the expressions of CD59, C3aR, and C5aR were extensively present in MUC1H ccRCC tissue samples and colocalized with PTX3. Finally, MUC1 expression was associated with an increased number of infiltrating mast cells, M2-macrophage, and IDO1+ cells, and a reduced number of CD8+ T cells. Taken together, our results suggest that expression of MUC1 can modulate the immunoflogosis in the ccRCC microenvironment by activating the classical pathway of the complement system and regulating the immune infiltrate, promoting an immune-silent microenvironment.

## 1. Introduction

Clear cell renal cell carcinoma (ccRCC) is the most common kidney tumor, and according to recent statistics, in the United States, 79,000 new cases were diagnosed and about 13,920 patients died from this disease in 2022 [1].

Many epidemiological studies have shown that obesity, diabetes, and other metabolic disorders can increase the risk of developing renal cancer [2,3,4]. In addition, in ccRCC, a particular metabolic signature has been described, characterized by a rerouting of the sugar metabolism toward the pentose phosphate pathway, significant accumulations of polyunsaturated fatty acids, and impaired mitochondrial activity [5,6,7,8,9,10,11].

An important goal in current research is to distinguish between different cancer subtypes based on the molecular characteristics that drive an aggressive phenotype, which can help predict clinical outcomes. Recently, we showed that mucin 1 (MUC1)-overexpressing ccRCCs were characterized by a specific metabolic modulation involving glucose and the lipid metabolism pathway [12]. In addition, higher levels of serum CA 15-3 (the soluble form of MUC1) were identified in patients with reduced cancer-specific (CSS) and progression-free survival (PFS) [12]. 

MUC1 is a cell membrane glycoprotein that is overexpressed and/or abnormally glycosylated in more than 70% of cancers. MUC1 is involved in many functions of cancer cells, such as cell adhesion, proliferation, migration, invasion, and metabolic reprogramming [13]. In addition, MUC1 acts as a modulator of chronic inflammation, although its role in regulating the tumor immune microenvironment (TME), especially in ccRCC, is poorly understood.

RCC has been defined as an immunogenic tumor with cytokine-based treatments as standard therapies before the introduction of antiangiogenic drugs and, more recently, immune checkpoint inhibitors (ICI) [14,15,16,17].

In this scenario, we previously showed that pentraxin-3 (PTX3), an acute-phase protein, was able to modulate the immunoflogosis in the ccRCC microenvironment through the activation of the classical pathway of the complement system and the release of pro-angiogenic factors [18,19].

In this study, we evaluated the in situ activation of the complement system (PTX3, C1q, C3aR, C5aR, CD59, and C5b-9) and characterized the immune cell infiltration (mast cells, macrophages, CD4, and CD8+ T cells) in correlation with the different expressions of MUC1 in a cohort of patients with ccRCC. 

## 2. Results

### 2.1. Gene Set Enrichment Analysis (GSEA) Shows Differences in Gene Expression Patterns between MUC1^H^ and MUC1^L^ Tumors

To compare the relative changes in the gene expression in ccRCC with high MUC1 expression (MUC1^H^) versus tumors with low MUC1 expression (MUC1^L^), gene expression data from the GSE15641 dataset were downloaded, and the samples were stratified by MUC1 expression. 

**Figure 1:**
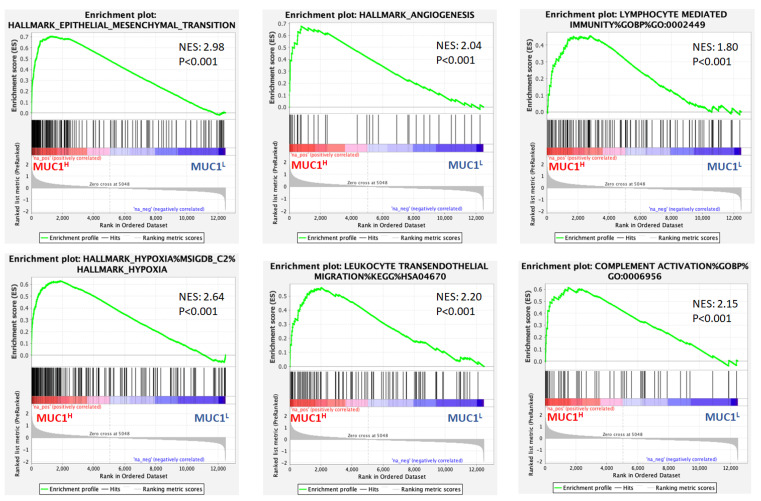
Gene set enrichment analysis (GSEA) of the GSE15641 dataset revealed that genes associated with MUC1 upregulation were significantly enriched in epithelial–mesenchymal transition, hypoxia, angiogenesis, complement system activation, and immune cell infiltration related processes. NES: normalized enrichment score.

Gene set enrichment analysis (GSEA) [20] demonstrated that MUC1^H^ ccRCC featured multiple enriched gene sets depicting epithelial–mesenchymal transition, hypoxia, angiogenesis, complement system activation, and immune cell infiltration related processes (Figure 1). 

It is well known that increased expression of MUC1 has been associated with the first two hallmarks, while there are few data about its role in modulating angiogenesis, complement system activation, and immune cell infiltration in ccRCC. In a previous study, we showed that pentraxin-3 (PTX3) can modulate the immunoflogosis in the ccRCC microenvironment through the activation of the classical pathway of the complement system (C1q) and the release of proangiogenic factors (C3a and C5a) [19]. Therefore, to evaluate the above findings according to MUC1 expression, we studied the PTX3 tissue expression and analyzed the activation of the complement system in tumor samples.

### 2.2. MUC1^H^ Renal Tumors Display an Altered Modulation of Immunoflogosis in TME

**Figure 2:**
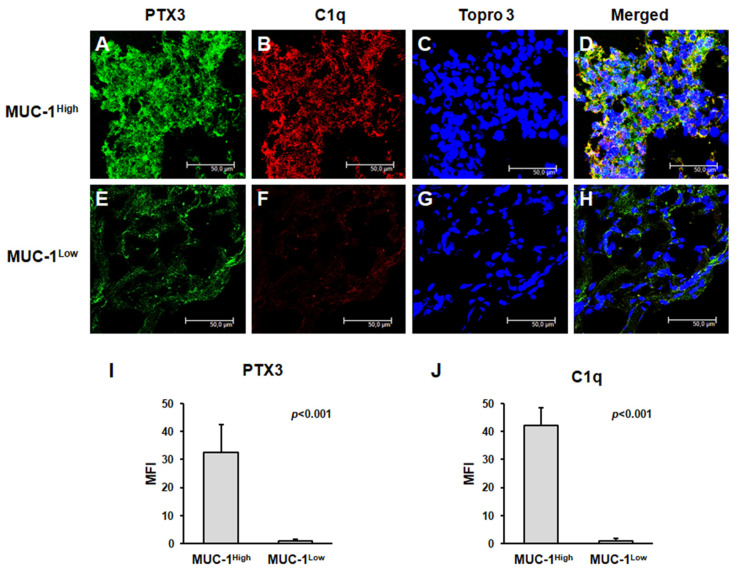
PTX3 protein expression and colocalization with C1q in neoplastic renal tissues from MUC1^H^ ccRCC (**A**–**D**) and MUC1^L^ ccRCC (**E**–**H**) by confocal microscopy and quantification of specific fluorescence (**I**,**J**).

PTX3 tissue expression was significantly higher in MUC1^H^ ccRCC (Figure 2A,I) than in the MUC1^L^ tumors (Figure 2E,I).

We then explored the activation of the complement system in both groups of ccRCC. Because PTX3 can activate the complement cascade through the classic pathway, we studied the deposition of C1q. Interestingly, C1q was extensively present in MUC1^H^ ccRCC tissue samples and colocalized with PTX3 (Figure 2A–D,J), while it was virtually absent in MUC1^L^ ccRCC tissue samples (Figure 2E–H,J).

**Figure 3:**
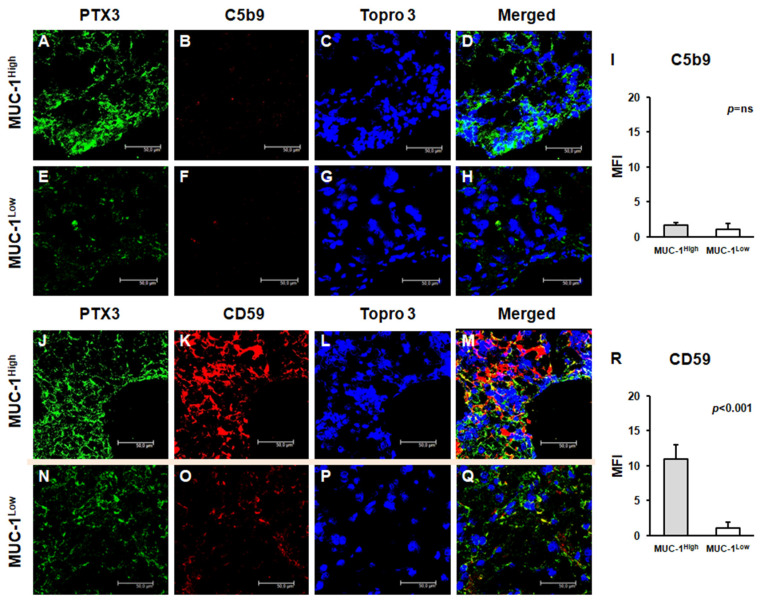
Expression of complement system factors’ C5b-9 (**A**–**H**) and CD59 (**J**–**Q**) and their colocalization with PTX3 in neoplastic renal tissues from MUC1^H^ ccRCC and MUC1^L^ ccRCC by confocal microscopy and quantification of specific fluorescence (**I**,**R**).

To validate the complete activation of the complement system, we next studied the tissue deposition of the terminal complement complex C5b-9, but the increased activation of the classical pathway did not correspond to an increased deposition of this complex. Indeed, C5b-9-specific immunofluorescence was substantially absent in the renal cancer tissue of both groups (Figure 3A–H), with no significant differences (Figure 3I). Subsequently, we evaluated the expression of CD59, a complement system regulatory protein that can inhibit the C5b-9 assembly. Notably, CD59 protein expression was markedly increased in MUC1^H^ ccRCC tissue samples (Figure 3J–M), while it was limited in MUC1L ccRCC (Figure 3N–Q), as shown by quantification of specific fluorescence (Figure 3R). 

**Figure 4:**
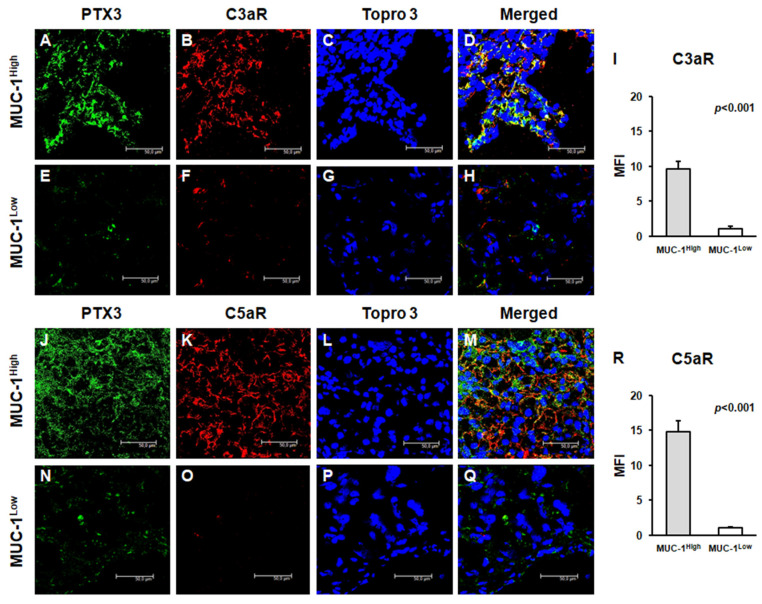
Expression of anaphylatoxin receptors C3aR and C5aR and their colocalization with PTX3 in neoplastic renal tissues from MUC1^H^ ccRCC (**A**–**H**) and MUC1^L^ ccRCC (**J**–**Q**) by confocal microscopy and quantification of specific fluorescence (**I**,**R**).

To evaluate the role of anaphylatoxins as potential soluble mediators modulating both cancer cell proliferation and neoplastic angiogenesis, we studied the expression of C3a and C5a receptors. The expression of both trans-membrane proteins was significantly increased in MUC1^H^ cancer tissues (Figure 4A–D,J–M), while it was significantly limited in MUC1^L^ ccRCC (Figure 4E–I,N–R). Moreover, the expressions of CD59, C3aR, and C5aR colocalized with PTX3 in MUC1^H^ cancer tissue (Figure 3M and Figure 4D,M, respectively).

### 2.3. TIMER and TISIDB Analyses Show MUC1 Modulation of Tumor-Infiltrating Immune Cells

**Figure 5:**
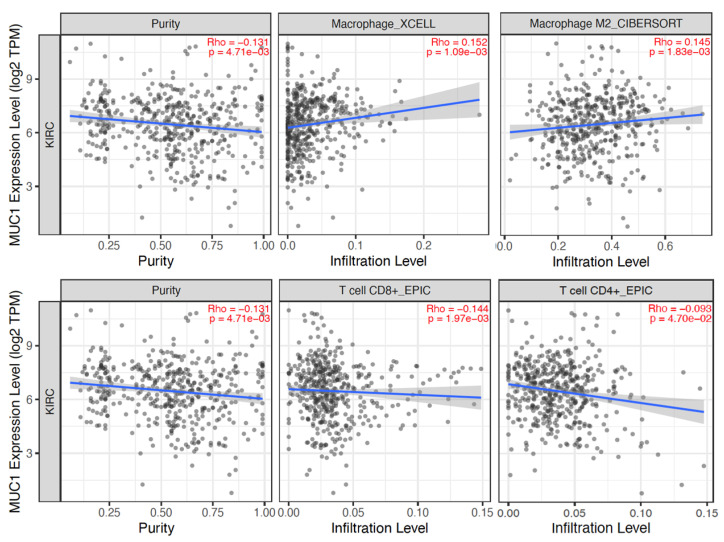
Correlation of MUC1 expression with tumor purity and infiltration level of indicated cell types in ccRCC samples. Data were downloaded from the TIMER web platform. Spearman’s correlation coefficients and *p* values are shown. TPM: transcript count per million reads.

The TIMER 2.0 (http://timer.cistrome.org, accessed on 10 January 2023) [21] and TISIDB (http://cis.hku.hk/TISIDB/index.php, accessed on 10 January 2023) [22] web resources were used to analyze the association between MUC1 expression and TME composition. TIMER analysis showed that MUC1 mRNA expression significantly correlated with macrophage (in particular M2 phenotype) and CD8+ T-cell enrichment, while MUC1 mRNA levels showed no significant association with CD4+ T-cell infiltration (Figure 5).

**Figure 6:**
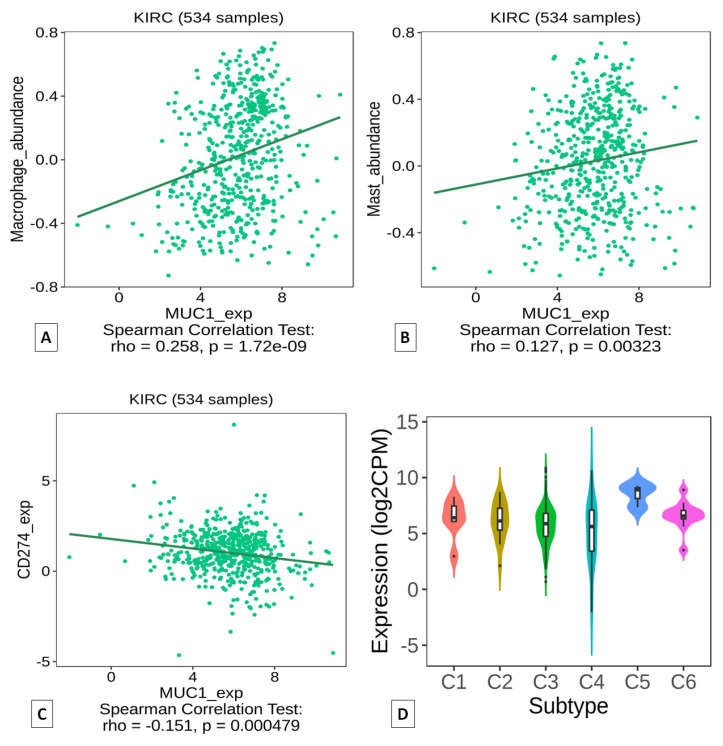
Correlation of MUC1 expression with infiltration level of macrophages (**A**) and mast cells (**B**) in ccRCC samples. Correlation between MUC1 and PD-L1 (CD274) expressions (**C**). Association between MUC1 expression and immune subtypes (**D**). Data were downloaded from the TISIDB web platform. Spearman’s correlation coefficients and *p* values are shown.

Analysis based on the TISIDB portal confirmed a positive correlation between MUC1 expression and macrophage abundance (Figure 6A), as well as a positive correlation between MUC1 and mast cell infiltration (Figure 6B). Conversely, a negative correlation was observed between MUC1 and PD-L1 (CD274) expression (Figure 6C). Finally, the analysis of the immune signature showed that MUC1^H^ ccRCC could mainly be classified as C5 (immunologically quiet) cancer immune subtype (Figure 6D).

### 2.4. MUC1 Expression Is Associated with Increased Angiogenesis and Subtype-Specific Immune Cell Infiltration

**Figure 7:**
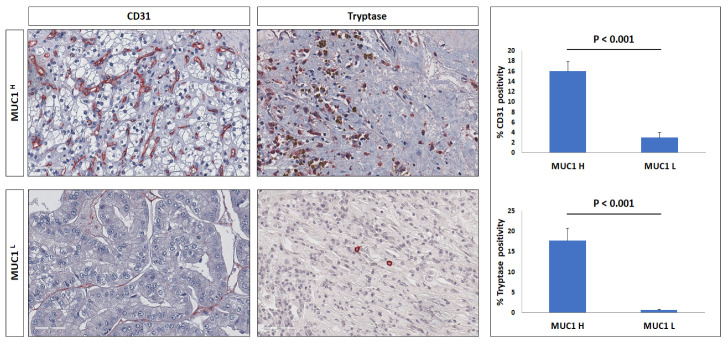
Immunohistochemical staining of CD31 microvessels and tryptase in MUC1^H^ ccRCC as compared with MUC1^L^ tumor.

As suggested by the GSEA results, MUC1 is implicated in other additional characteristics of tumor biology, including induction of angiogenesis and immune cell infiltrate modulation. To investigate the angiogenic response, we evaluated the vascular density through CD31 immunostaining in MUC1^H^ versus MUC1^L^ histological specimens derived from ccRCC patients. We found a significantly increased number of CD31+ microvessels in MUC1^H^ ccRCC samples compared with those in MUC1^L^ tumors (Figure 7).

Next, ccRCC specimens were immunostained for tryptase, CD68, and CD163 to estimate mast cells, total tumor-associated macrophages (TAMs), and M2-TAMs subpopulations, separately. 

Much of the evidence suggests that mast cells can promote tumor progression and angiogenesis [23,24]; thus, we evaluated the infiltration of tryptase-positive cells in the tumor microenvironment according to MUC1 expression. MUC1^H^ ccRCC showed an increased number of these cells compared with MUC1^L^ cancer tissues (Figure 7).

**Figure 8:**
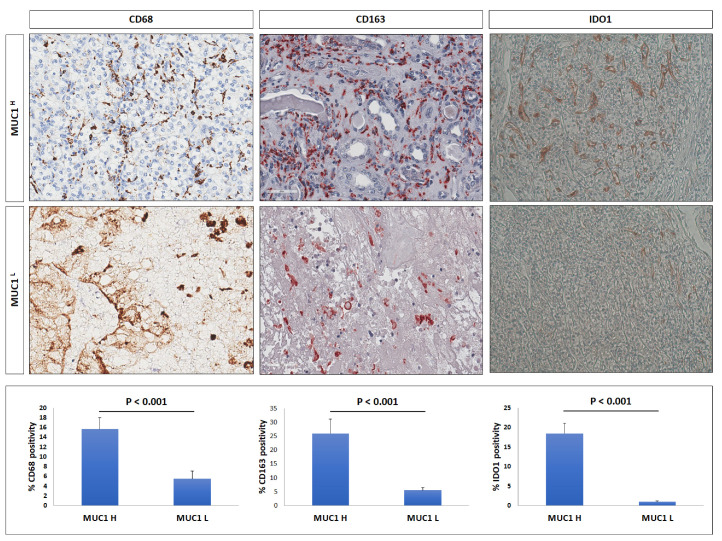
Immunohistochemical staining of CD68, CD163, and IDO1 in MUC1^H^ ccRCC compared with those in MUC1^L^ tumors.

To estimate macrophage infiltration and the status of polarization, we studied CD68 and CD163 expression. IHC showed an increased expression of these markers in MUC1^H^ ccRCC compared with in MUC1^L^ tumors (Figure 8). 

**Figure 9:**
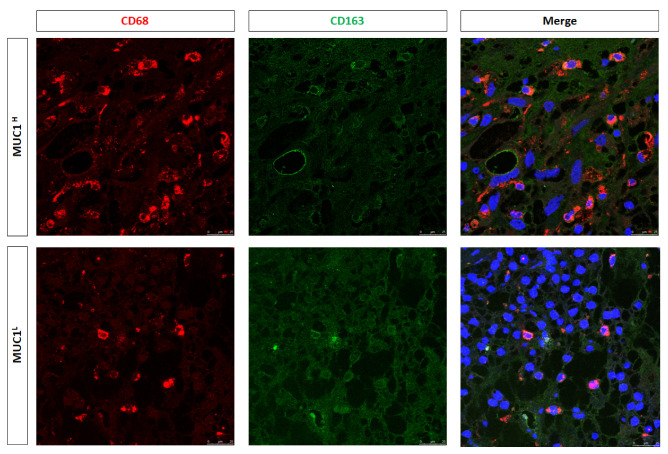
Expression of CD68 and CD163 and their colocalization in neoplastic renal tissues from MUC1^H^ ccRCC and MUC1^L^ tumors.

In addition, immunofluorescence and confocal laser scanning microscopy demonstrated the colocalization of the two markers (Figure 9), confirming the M2 polarization status of macrophages.

**Figure 10:**
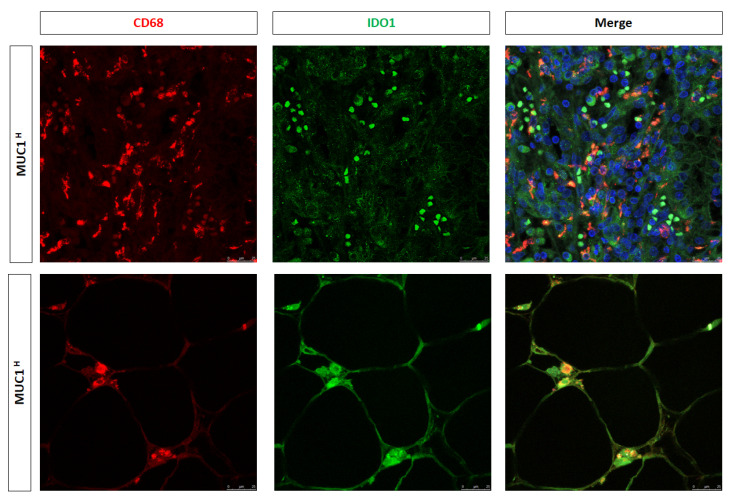
Expression of CD68 and IDO1 and their colocalization in neoplastic renal tissues from MUC1^H^ ccRCC and MUC1^L^ tumors.

In a previous study [25], we showed that activation of the kynurenine (KYN) pathway promoted renal cancer cells’ survival, migration, and chemoresistance and that increased accumulation of KYN in ccRCC was sustained by IDO1+ macrophages. To study the above findings in MUC1-expressing tumors, we performed immunohistochemistry for IDO1 and immunofluorescence for CD68/IDO1 coexpression. Immunostaining demonstrated a stronger signal in MUC1^H^ tumors (Figure 8), and confocal laser scanning microscopy images confirmed a colocalization for both IDO1 and CD68 markers in infiltrating cells, demonstrating that the IDO1-positive cells were macrophages (Figure 10).

Considering the role of cancer-associated mucins in modulating immune cell response and TME infiltration, we analyzed the presence of CD8+ and CD4+ T cells, and PD-L1 immunostaining in the ccRCC microenvironment.

**Figure 11:**
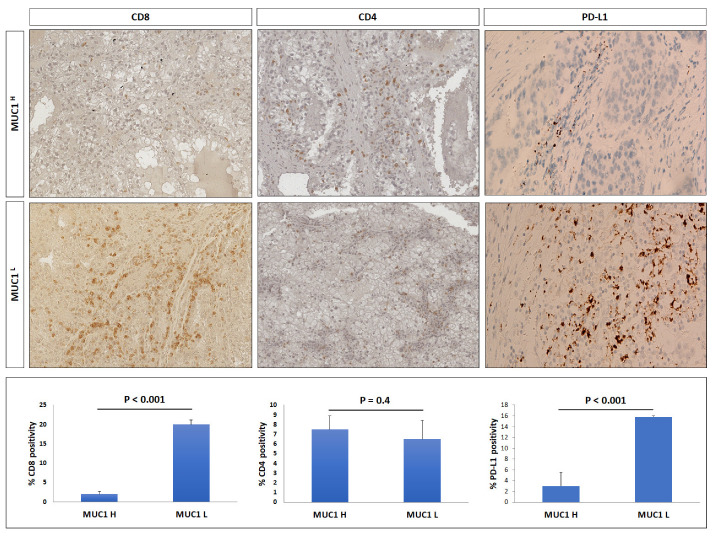
Immunohistochemical staining of CD8, CD4, and PD-L1 in MUC1^H^ ccRCC compared with MUC1^L^ tumor.

As concerns CD8-positive cells, their number in MUC1^H^ tumors was very low compared to MUC1^L^ specimens, whereas the number of CD4-positive lymphocytes did not show any significant variation in either group (Figure 11).

Finally, we evaluated the role of the MUC1 in modulating the immune checkpoint ligand PD-L1 expression in ccRCC. Our findings showed that MUC1^H^ tumors had reduced expression levels of PD-L1 (Figure 10), and these findings were in accordance with the TISIDB analysis results.

### 2.5. MUC1 Soluble Form (Serum CA15-3) Correlates with PTX3 Serum Levels, KYN-to-Tryptophan Ratio (KTR), and Other Systemic Inflammation Biomarkers

We previously showed that serum PTX3 and MUC1 soluble form, also known as CA15-3, could serve as a circulating biomarker to identify ccRCC patients with poor outcomes [12,26]. Moreover, a previous study showed that increased KTR, a clinical marker of IDO1 activity, is a negative prognostic factor for cancer-specific and progression-free survival [25].

**Figure 12:**
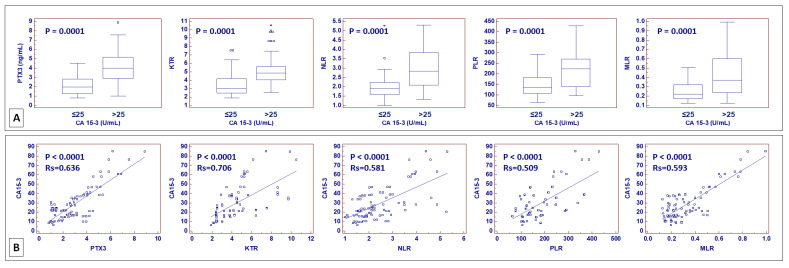
Box-and-whisker plots of pentraxin 3 (PTX3) serum levels, kynurenine-to-tryptophan ratio (KTR), neutrophil-to-lymphocyte ratio (NLR), platelet-to-lymphocyte ratio (PLR), and monocyte-to-lymphocyte ratio (MLR), stratified according to CA15-3 values (≤25 vs. >25 U/mL) (**A**). Scatter diagrams with regression line for correlation analysis between CA15-3 and PTX3, KTR, NLR, PLR, and MLR (**B**).

In addition, in recent years, different parameters originating from routine complete blood counts have been investigated as potential biomarkers of cancer-associated systemic inflammation. These include the neutrophil-to-lymphocyte ratio (NLR), platelet-to-lymphocyte ratio (PLR), and monocyte-to-lymphocyte ratio (MLR) [27,28,29,30,31,32]. To evaluate their correlation, CA15-3, serum PTX3, KTR, NLR, PLR, and MLR levels were preoperatively measured in patients who underwent nephrectomy for ccRCC at our institution. Patients with an abnormal value of CA15-3 also had increased serum levels of PTX3 (*p* < 0.001) and increased KTR (*p* < 0.001), NLR (*p* < 0.001), PLR (*p* < 0.001), and MLR (*p* < 0.001) (Figure 12A). Spearman’s test confirmed these results, showing a positive correlation between CA15-3 and PTX3, KTR, NLR, PLR, and MLR (Figure 12B).

## 3. Discussion

In recent years, the multiomics characterization of ccRCC has resulted in the identification of specific molecular pathways that play a critical role in tumor pathogenesis and progression [5,6,7,8,9,10,33]. In addition, a better understanding of the molecular heterogeneity associated with the tumor microenvironment has revolutionized the therapy of patients with metastatic disease owing to the introduction of ICI [34,35,36].

Renal cell carcinoma is typically characterized by intratumor heterogeneity (ITH), and the identification of the mechanisms involved in this process represents a crucial issue also in the clinical setting because ITH is an important factor associated with treatment failure [35]. 

In a recent study, we showed that MUC1-expressing ccRCC is characterized by a specific signature consisting of impaired glucose and lipid metabolism [12]. In addition, MUC1 expression was associated with increased renal cancer cell proliferation and migration and had a prognostic role in patients affected by ccRCC [12]. 

To better define the role of MUC1 in additional renal-cancer-associated processes, we performed a GSEA using the Jones cohort (GSE15641) and stratified the patients according to MUC1 expression. Interestingly, this analysis revealed a previously unappreciated significance of MUC1 in modulating complement system activation.

In a previous study, we demonstrated that increased expression of PTX3 in ccRCC was associated with the partial activation of the complement system with an overexpression of C1q, C3aR, and C5aR [19]. In the current study, we found that C1q deposition was extensively present in MUC1^H^ ccRCC tissue samples and colocalized with PTX3. Moreover, in accordance with our previous results, the activation of the classical pathway did not correspond to an increased deposition of the terminal complex C5b-9, and this finding was associated with the increased expression of CD59, a complement system regulatory protein that prevents C5b-9 assembly. MUC1^H^ tumors showed overexpression of the receptors of the anaphylatoxins C3a (C3aR) and C5a (C5aR).

These proteins modify the immunological makeup of the TME, can activate mast cells and M2 TAMs, and are associated with increased tumor growth [37]. 

Angiogenesis is a hallmark of RCC and plays a pivotal role in the different phases of ccRCC progression [38,39]. We found that MUC1^H^ tumors were characterized by a higher microvascular density in association with an increased number of mast cells. Tuna et al. observed a significant correlation between microvessel density and mast cell infiltration in RCC [40]. Mast cells are tissue-resident cells that actively participate in the tumor-associated angiogenic switch and shape the immune microenvironment [41,42]. Our findings were confirmed by a TISIDB analysis that showed a positive correlation between MUC1 expression and mast cell abundance. 

As concerns macrophages, we observed an increased number of CD68+CD163+ and CD68+IDO1+ cells in MUC1^H^ tumor samples, revealing that TAMs were M2-polarized and able to produce KYN. These findings support our previous results that showed the accumulation of this metabolite in MUC1^H^ versus MUC1^L^ ccRCC [25]. The increased levels of KYN could contribute to an aggressive phenotype and CD8+ T-cell depletion observed in MUC1H tumors. 

Clear cell RCC has been classically identified as an immunotherapy-responsive tumor. The cytokine-based therapeutic protocols have been the standard of care in patients with advanced disease before the introduction of antiangiogenic drugs [43,44]. Recently, the progressive use of ICI has proved to be extremely effective even in this type of tumor, and combination therapies are becoming more widespread [45]. However, it is known that the efficacy of ICI is mainly predicted by PD-1/PD-L1 expression levels in association with tumor mutational burden, tumor-infiltrating lymphocytes, and other immune-related factors. In this scenario, our study showed that PD-L1 (CD274) expression was reduced in MUC1^H^ ccRCC, as predicted by the TISIDB analysis.

Finally, we analyzed the correlation between CA15-3—a circulating biomarker derived from MUC1—and different serum parameters associated with cancer-related systemic inflammation. In all cases, the correlation analyses demonstrated a positive relationship between CA15-3 serum levels and the other markers. 

Taken together, our results suggest that MUC1-expressing tumors can be identified as an immune-silent subgroup of ccRCC, characterized by low immune infiltration, high microvessel density, high M2-TAM response, and altered metabolism [46]. Moreover, in accordance with our findings, the analysis of the immune signature of the TCGA KIRC cohort showed that MUC1^H^ ccRCC can mainly be classified as a C5 (immunologically quiet) cancer immune subtype [47].

In conclusion, this study suggests that expression of MUC1 can modulate the immunoflogosis in the ccRCC microenvironment by activating the classical pathway of the complement system and regulating the immune infiltrate, promoting an immune-silent microenvironment. In particular, MUC1-expressing ccRCC is characterized by altered metabolism, high microvessel density, high M2-TAM response, low immune infiltration, and low expression of PD-L1. This particular phenotype would make these tumors more responsive to antiangiogenic therapies rather than ICI.

## 4. Materials and Methods

### 4.1. Study Population, Tissue Collection, and Circulating Biomarkers Evaluation

An investigation was conducted in accordance with the Declaration of Helsinki and national and international guidelines and approved by the authors’ institutional review board. Written informed consent to take part was given by all participants. The primary renal tumor (n = 36) was collected from patients who underwent nephrectomy for ccRCC (Supplementary Table S1). For circulating biomarkers evaluation, serum CA15-3 (normal values: 0–25 U/mL), PTX3 (normal values: 0–2 ng/mL), kynurenine-to-tryptophan ratio (KTR), neutrophil-to-lymphocyte ratio (NLR), platelet-to-lymphocyte ratio (PLR), and monocyte-to-lymphocyte ratio (MLR) were preoperatively measured in a cohort of 86 consecutive patients who underwent radical or partial nephrectomy for ccRCC at our institution. The pathological and clinical characteristics of these patients are summarized in Supplementary Table S2. Measurement of serum CA15-3 was performed with electrochemiluminescence immunoassay (ECLIA) on a fully automated Roche Cobas 8000 analyzer (Roche Diagnostics GmbH, Mannheim, Germany). Measurement of PTX3 was performed with sandwich ELISA on an automated platform (DSX, Technogenetics srl, Milano, Italy). Serum tryptophan (TRP) and kynurenine (KYN) levels were quantitatively determined with a tryptophan ELISA Kit (Abnova Corporation, Taipei, Taiwan) and human kynurenine ELISA Kit (Cusabio Biotech, Wuhan, China). 

### 4.2. Gene Set Enrichment Analysis (GSEA)

Gene expression data from the GSE15641 dataset were used and GSEA was run to determine the statistically enriched pathways in the ccRCC dataset [20]. 

### 4.3. Immunohistochemistry

For immunohistochemical evaluation, the following antibodies were used: mouse monoclonal anti-MUC1 (NB-120-22711, Novus Biologicals, Littleton, CO, USA), mouse monoclonal anti-tryptase (NB-100-64820, Novus Biologicals, Littleton, CO, USA), rabbit polyclonal anti-CD31 (ab28364, Abcam, Cambridge, UK), mouse monoclonal anti-CD68 (NCL-CD68-KP1, Novocastra Laboratories Ltd., Newcastle, UK), mouse monoclonal anti-CD163 (NCL-L-CD163, Novocastra Laboratories Ltd., Newcastle, UK), and mouse monoclonal anti-IDO1 (ab156787, Abcam, Cambridge, UK), diluted according to the respective datasheet indications. 

### 4.4. Indirect Immunofluorescence and Confocal Laser Scanning Microscopy

A double-label immunofluorescence was performed to evaluate the expressions of PTX-3, C1q, C5b-9, CD59, C3aR, C5R1, CD68, IDO1, and CD163 and their eventual co-localizations. The following primary antibodies were used: rat monoclonal IgG2a anti-PTX-3 antibody (clone MNB4, Abcam, Cambridge UK); mouse monoclonal IgG2b anti-C1q (clone JL-1; Abcam); mouse monoclonal IgG2a anti-C5b-9 (clone aE11; Abcam); rabbit polyclonal IgG anti-CD59 (Sigma-Merck KGaA, Darmstadt, Germany); rabbit polyclonal IgG anti-C3aR (Abcam); mouse monoclonal IgG2a anti-C5R1/CD88 (clone P12/1; Abcam); rabbit polyclonal IgG anti-IDO1 (Novus Biologicals); mouse monoclonal IgG1 anti-CD68 (clone KP1; Abcam); and mouse monoclonal IgG1 anti-CD163 (clone RM3/1; Santa Cruz Biotechnology, Dallas, TX, USA). To stain the nuclei, samples were incubated with TO-PRO diluted 1:5000 in PBS pH 7.4 (Invitrogen-Molecular Probe, Thermo Fisher, Waltham, MA, USA). 

Additional details regarding the experimental procedures are provided in the Supplementary Materials and Methods.

## Data Availability

The datasets generated and/or analyzed during the current study are available in the GEO repository: Accession number GSE15641.

## Supplementary Materials

The following supporting information can be downloaded at: https://www.mdpi.com/article/10.3390/ijms24054814/s1.
