# Supplementary material for: MUC1 Expression Affects the Immunoflogosis in Renal Cell Carcinoma Microenvironment through Complement System Activation and Immune Infiltrate Modulation
Source: Int J Mol Sci. 2023 Mar 2;24(5):4814. doi: 10.3390/ijms24054814 (PMC10003656; doi:10.3390/ijms24054814)
Supplement: Supplementary file 1 [file ijms-24-04814-s001.zip › ijms-2223402-Supplementary Materials and Methods.pdf]

Supplementary Materials and Methods

Study population, tissue collection, and circulating biomarkers evaluation

Investigation has been conducted in accordance with the Declaration of Helsinki and national and international guidelines and has been approved by the authors' institutional review board. Written informed consent to take part was given by all participants. Primary renal tumor (n = 36) was collected from patients who underwent nephrectomy for ccRCC (Supplementary Table 1). Patients with metabolic diseases and/or an estimated glomerular filtration rate (MDRD equation) < 60 mL/min/1.73 m^2 were excluded from the study.

As circulating biomarkers evaluation, serum CA15-3 (normal values: 0–25 U/mL), PTX3 (normal values: 0-2 ng/mL), kynurenine-to-tryptophan ratio (KTR), neutrophil-to-lymphocyte ratio (NLR), platelet-to-lymphocyte ratio (PLR) and monocyte-to-lymphocyte ratio (MLR), were preoperatively measured in a cohort of 86 consecutive patients who underwent radical or partial nephrectomy for ccRCC at our institution. Patients with known causes of elevated levels of CA15-3 were excluded (namely, patients with other cancers, sarcoidosis, endometriosis, tuberculosis, systemic lupus erythematosus, liver and breast diseases). Pathological and clinical characteristics of these patients are summarized in Supplementary Table 2.

Measurement of serum CA15-3 was performed by electrochemiluminescence immunoassay (ECLIA) on a fully automated Roche Cobas 8000 analyzer (Roche Diagnostics GmbH, Mannheim, Germany).

Measurement of PTX3 was performed by sandwich ELISA, on automated platform DSX (Technogenetics srl, Milano, Italy).

Serum tryptophan (TRP) and kynurenine (KYN) levels were quantitatively determined by Tryptophan ELISA Kit (Abnova Corporation, Taipei, Taiwan) and Human kynurenine ELISA Kit (Cusabio Biotech , Wuhan, China). These assays employed the competitive inhibition enzyme immunoassay technique. The microtiter plates provided in these kits have been pre-coated with an antigen. Standards or samples or controls were added to the appropriate microtiter plate wells with antibody specific for TRP or KYN and Horseradish Peroxidase (HRP) conjugated goat-anti-rabbit antibody using tetramethyl benzidine as a substrate. The reaction was monitored at 450 nm. Quantification of unknown samples was performed by comparing their absorbance with a standard curve prepared with known standards. All samples were run in duplicate.

Gene Set Enrichment Analysis (GSEA)

To identify genes associated with high versus low MUC1 gene expression, we used the gene expression data from the GSE15641 dataset (23 normal kidney and 32 ccRCC). The patients were stratified by MUC1 expression, and then a rank file for each expressed gene by the log2 fold change in high MUC1 samples over low MUC1 samples was generated. Next, we ran a gene set enrichment analysis (GSEA) to determine which pathways were statistically enriched across the ccRCC dataset [20]. The normalized enrichment score (NES) was used to evaluate the extent and direction of enrichment of each pathway.

Immunohistochemistry

Serial sections 4 μm-thick were cut from formalin-fixed paraffin-embedded tissue and mounted on poly-L-lysine-coated glass slides. Sections were deparaffinized and rehydrated through xylenes and graded alcohol series.

Slides were subjected to specific epitope demasking by microwave treatment at 700 W in citrate buffer (0.01 M, pH 6.0). After antigen retrieval, the

tissue samples were incubated for 10 minutes with 3% H2O2 to block endogenous peroxidase activity. Sections were blocked with Protein Block Serum-Free (Dako) at room temperature for 10 minutes and then incubated with mouse monoclonal anti-MUC1 (NB-120-22711, Novus Biologicals, Littleton, CO), mouse monoclonal antitryptase (NB-100-64820, Novus Biologicals, Littleton, CO), rabbit polyclonal anti-CD31 (ab28364, Abcam, Cambridge, UK), mouse monoclonal anti-CD68 (NCL-CD68-KP1, Novocastra Laboratories Ltd, Newcastle, UK), mouse monoclonal anti-CD163 (NCL-L-CD163, Novocastra Laboratories Ltd, Newcastle, UK) and mouse monoclonal anti-IDO1 (ab156787, Abcam, Cambridge, UK), diluted according to the respective datasheet indications. Binding of the secondary biotinylated antibody was detected using the Dako Real EnVision Detection System, Peroxidase/DAB kit (Dako, Agilent, Santa Clara, US), according to the manufacturer's instructions. Sections were counterstained with Mayer's haematoxylin (blue) and mounted with glycerol (Dako Cytomation). Negative controls were obtained by incubating serial sections with the blocking solution and then omitting the primary antibodies. Sections from both experimental groups ($MUC1^H$and $MUC1^L$), were scanned using the whole-slide morphometric analysis scanning platform Aperio Scanscope CS (Leica Biosystems, Nussloch, Germany). All the slides were scanned at the maximum available magnification (40x) and stored as digital high resolution images on the workstation associated with the instrument. Digital slides were inspected with Aperio ImageScope v.11 software (Leica Biosystems, Nussloch, Germany) at 20X magnification and 10 fields with an equal area were selected for the analysis at 40X magnification. The protein expression was assessed with the Positive Pixel Count algorithm embedded in the Aperio ImageScope software and reported as positivity percentage, defined as the number of positively stained pixels on the total pixels in the image.

Indirect immunofluorescence and confocal laser scanning microscopy

A double-label immunofluorescence was performed to evaluate the expression of PTX-3, C1q, C5b-9, CD59, C3aR, C5R1, CD68, IDO1 and CD163, and their eventual co-localization. To this purpose we employed the following primary antibodies: rat monoclonal IgG2a anti-PTX-3 antibody (clone MNB4, Abcam, Cambridge UK), mouse monoclonal IgG2b anti-C1q (clone JL-1; Abcam); mouse monoclonal IgG2a anti-C5b-9 (clone aE11; Abcam); rabbit polyclonal IgG anti-CD59 (Sigma-Merck KGaA, Darmstadt, Germany); rabbit polyclonal IgG anti-C3aR (Abcam); mouse monoclonal IgG2a anti-C5R1/CD88 (clone P12/1; Abcam); rabbit polyclonal IgG anti-IDO1 (Novus Biologicals); mouse monoclonal IgG1 anti-CD68 (clone KP1; Abcam); mouse monoclonal IgG1 anti-CD163 (clone RM3/1; Santa Cruz Biotechnology).

After paraffin removal, tissue sections were incubated at 4°C over night with a mixture of primary antibodies diluted 1:100 in PBS pH 7.4. The immune complexes were detected using the Alexa-Fluor 488 goat anti-rat, 546 goat anti-mouse IgG, 546 goat anti-rabbit IgG (all from Alexa, Thermo Fisher, Waltham, MA), Alexa Fluor 555 goat anti-mouse IgG (Molecular Probes), goat anti-rabbit IgG FITC (Novus Biologicals) and Alexa Fluor 488 goat anti-mouse (Molecular Probes).

After washing in PBS (3x5') the sections and the negative control were incubated 1h at room temperature with goat anti-rat IgG 488 and goat anti-mouse IgG 546 or goat anti-rabbit IgG 546; with Alexa Fluor 555 goat anti-mouse and goat anti-rabbit IgG FITC or Alexa Fluor 488 goat anti-mouse, as appropriate. All secondary antibodies were used at a dilution of 1:250.

To stain the nuclei, after washing in PBS pH 7.4 (3×5') samples were incubated with TO-PRO diluted 1:5000 in PBS pH 7,4 (Invitrogen-Molecular

Probe, Thermo Fisher, Waltham, MA). The slides were mounted in Gel Mount (Sigma) and sealed.

Specific fluorescence was evaluated by confocal microscopy using the Leica TCS SP5 (Leica, Wetzlar, Germany) equipped with argon-krypton (488 nm), green-neon (543 nm), and helium-neon (633 nm) lasers.
