# Supplementary material for: MUC1 Expression Affects the Immunoflogosis in Renal Cell Carcinoma Microenvironment through Complement System Activation and Immune Infiltrate Modulation
Source: Int J Mol Sci. 2023 Mar 2;24(5):4814. doi: 10.3390/ijms24054814 (PMC10003656; doi:10.3390/ijms24054814)
Supplement: Supplementary file 1 [file ijms-24-04814-s001.zip › Supplementary Table S1.pdf]

Variable	n=36
Age (years)<br>median<br>range	 61<br>27-82
Gender<br>Male<br>Female	 23 (63.8%)<br>13 (36.2%)
Dimensions (cm)<br>median<br>range	 5.0<br>3 -10
Pathological stage<br>pT1<br>pT2<br>pT3	 22 (62%)<br>9 (25%)<br>5 (13%)
pN+	7 (19.4%)
cM+	3 (8.3%)
Fuhrman grade<br>G1-2<br>G3-4	 24 (66.7%)<br>12 (33.3%)

Table S1: Clinical and pathological characteristics of tissues collected from patients who underwent radical or partial nephrectomy for ccRCC. These specimens were used for Immunohistochemistry, indirect immunofluorescence and confocal laser scanning microscopy.
