# Supplementary material for: MUC1 Expression Affects the Immunoflogosis in Renal Cell Carcinoma Microenvironment through Complement System Activation and Immune Infiltrate Modulation
Source: Int J Mol Sci. 2023 Mar 2;24(5):4814. doi: 10.3390/ijms24054814 (PMC10003656; doi:10.3390/ijms24054814)
Supplement: Supplementary file 1 [file ijms-24-04814-s001.zip › Supplementary Table S2.pdf]

Variable	n=86
**Age (years)**	
**median**	61
**95% CI**	59-65
**Gender**	
**Male**	53 (62%)
**Female**	33 (38%)
**Dimensions (cm)**	
**median**	4.5
**95% CI**	4.0 - 5.0
**Pathological stage**	
**pT1a**	33 (38%)
**pT1b**	24 (28%)
**pT2**	14 (16%)
**pT3**	12 (14%)
**pT4**	3 (4%)
**pN+**	6 (7%)
**cM+**	5 (6%)
**Fuhrman grade**	
**G1-2**	52 (61%)
**G3-4**	34 (39%)
**CA15-3 (U/mL)**	
**Median**	23.9
**95% CI**	21.2 – 28.3
**range**	6.20 – 85.0
**PTX3 (ng/mL)**	
**Median**	2.73
**95% CI**	2.33 – 3.17
**range**	0.79 – 8.87
**KTR**	
**Median**	4.0
**95% CI**	3.35 – 4.51
**range**	1.90 – 10.5
**NLR**	
**Median**	2.12
**95% CI**	1.91 – 2.46
**range**	1.01 – 5.29

**PLR**	
**Median**	162.6
**95% CI**	136.7 – 185.4
**range**	64.0 – 428.3
**MLR**	
**Median**	0.26
**95% CI**	0.23 – 0.32
**range**	0.12 – 0.99

Table S2: Clinical and pathological characteristics of patients enrolled for serum biomarkers evaluation. KTR: kynurenine-to-tryptophan ratio; NLR: neutrophil-to-lymphocyte ratio; PLR: platelet-to-lymphocyte ratio; MLR: monocyte-to-lymphocyte ratio.
